# Computational analysis of non-coding RNAs in Alzheimer's disease

**DOI:** 10.6026/97320630015351

**Published:** 2019-05-15

**Authors:** Ghulam Md Ashraf, Magdah Ganash, Alexiou Athanasios

**Affiliations:** 1 King Fahd Medical Research Center, King Abdulaziz University, P.O. Box 80216, Jeddah 21589, Saudi Arabia; 2 Department of Biology, Faculty of Science, King Abdulaziz University, Jeddah, Saudi Arabia; 3 Novel Global Community Educational Foundation, 7 Peterlee Place, Hebersham, NSW 2770, Australia; 3 AFNP Med, Austria

**Keywords:** Alzheimer's disease, BACE1-AS, NAT-Rad18, 17A, hnRNP Q, long noncoding RNAs, RAD18, secondary structure prediction, strict β-turns, structural alignment

## Abstract

Latest studies have shown that Long Noncoding RNAs corresponds to a crucial factor in neurodegenerative diseases and next-generation therapeutic targets. A wide range of advanced computational methods for the analysis of Noncoding RNAs mainly includes the prediction of RNA and miRNA structures. The problems that concern representations of specific biological structures such as secondary structures are either characterized as NP-complete or with high complexity. Numerous algorithms and techniques related to the enumeration of sequential terms of biological structures and mainly with exponential complexity have been constructed until now. While BACE1-AS, NATRad18, 17A, and hnRNP Q lnRNAs have been found to be associated with Alzheimer's disease, in this research study the significance of the most known β-turn-forming residues between these proteins is computationally identified and discussed, as a potentially crucial factor on the regulation of folding, aggregation and other intermolecular interactions.

## Background

Noncoding RNAs (ncRNAs) play important roles in many biological mechanisms offering to the researcher's opportunities for efficient biomarkers' detection and disease diagnosis, treatment, prognosis and prevention 1-3. While only 1.5% of the whole genome is corresponding to protein-coding genes 1, various Long Noncoding RNAs (lncRNAs) such as BACE1-AS are closely related to the Alzheimer's disease (AD) 4-6, modulating Aβ formation or impacting apoptosis 7-9. Beta-site Amyloid Precursor Protein Cleaving Enzyme 1 - Antisense Transcript (BACE1-AS) enhances BACE1 mRNA stability by protecting it from degradation 9, concluding to a highly correlation with AD development or progression 5,10-12 as well as lncRNA-17A which play a significant role in Gamma-Aminobutyric Acid Type B Receptor Subunit 2 (GABABR2) signaling and Aβ production 7,9. Additionally, heterogeneous nuclear Ribonucleoprotein Q (hnRNPs) family assist in controlling the maturation of newly formed heterogeneous nuclear RNAs (hnRNAs/pre-mRNAs) into messenger RNAs (mRNAs), stabilize mRNA during their cellular transport and control their translation 13, affecting the dendritic development 14-19. Latest studies also reveal the role of Postreplication repair protein RAD18 (NAT-Rad18) in AD by affecting the DNA repair system, leading to apoptosis and neurodegenration 7. In contrast to protein folding programs, where the tertiary structure is predicted, the majority of the currently available RNA M-folding algorithms concentrate on the secondary structure of the RNA structure. Current RNA prediction algorithms have a polynomial runtime of O(n3) where n is the sequence length. Still, the mere knowledge of the secondary structure can be misleading, as two similar tertiary structures can have different secondary structures 20. The problems that concern representations of certain biological structures such as secondary structures are either characterized as NP-complete or with high complexity. The incompleteness of the corresponding theories contributes to a kind of hybrid problem, where data mining, statistical analysis, biological interpretation, and computational techniques must interact in different phases, in order to produce a solution. Numerous algorithms and techniques related to the enumeration of sequential terms of biological structures and mainly with exponential complexity have been constructed through their bijection with alternative representations such as energy models, plane trees and Motzkin numbers, non-crossing set partitions, Motzkin paths and Dyck paths 21. In contrast to protein folding programs, where the tertiary structure is predicted, the majority of the currently available RNA M-folding algorithms concentrate on the secondary structure of the RNA structure. The first reason for this difference is a pragmatic one. Current RNA prediction algorithms have a polynomial runtime of O(n3) where n is the sequence length. This is fast enough to allow genome-wide analysis on current off-the-shelf computers. The consideration of the tertiary structure, however, leads to a super polynomial-runtime impeding any large-scale application 22. The second reason is related to the kinetic of RNA folding. Secondary structures form first, leading to a set of loops and helices, which once formed, interact to yield the tertiary structure. As a consequence, the determination of the tertiary structure depends strongly on the secondary structure 23. Still, the mere knowledge of the secondary structure can be misleading, as two similar tertiary structures can have different secondary structures 20.

## Methodology

Latest studies have already revealed the correlation between specific lncRNAs to AD pathologies and lesions in brain regions like the middle temporal gyrus, the prefrontal cortex, the striatum the cerebellum and the hippocampus and other CNS related disorders 8, 24-27. The secondary structures of four proteins related to AD have been examined in this study BACE1, Rad18, GABABR2 and hnRNPQ targeted from the corresponding lnRNAs BACE1-AS, NAT-Rad18, 17A and hnRNP Q 28. A protein statistics-analysis was initially executed with the QIAGEN CLC Main Workbench (supplementary material available with authors). For the computational analysis the sequences 6EJ3(BACE1_HUMAN), 4F12(GABABR2_HUMAN), 4UX8 (hnRNPQ_HUMAN), 2Y43 (RAD18_HUMAN) were imported from the Protein Databank, avoiding the use of prediction methods for the identification of secondary elements in order to reduce additional errors.

## Results

**Figure 1:**
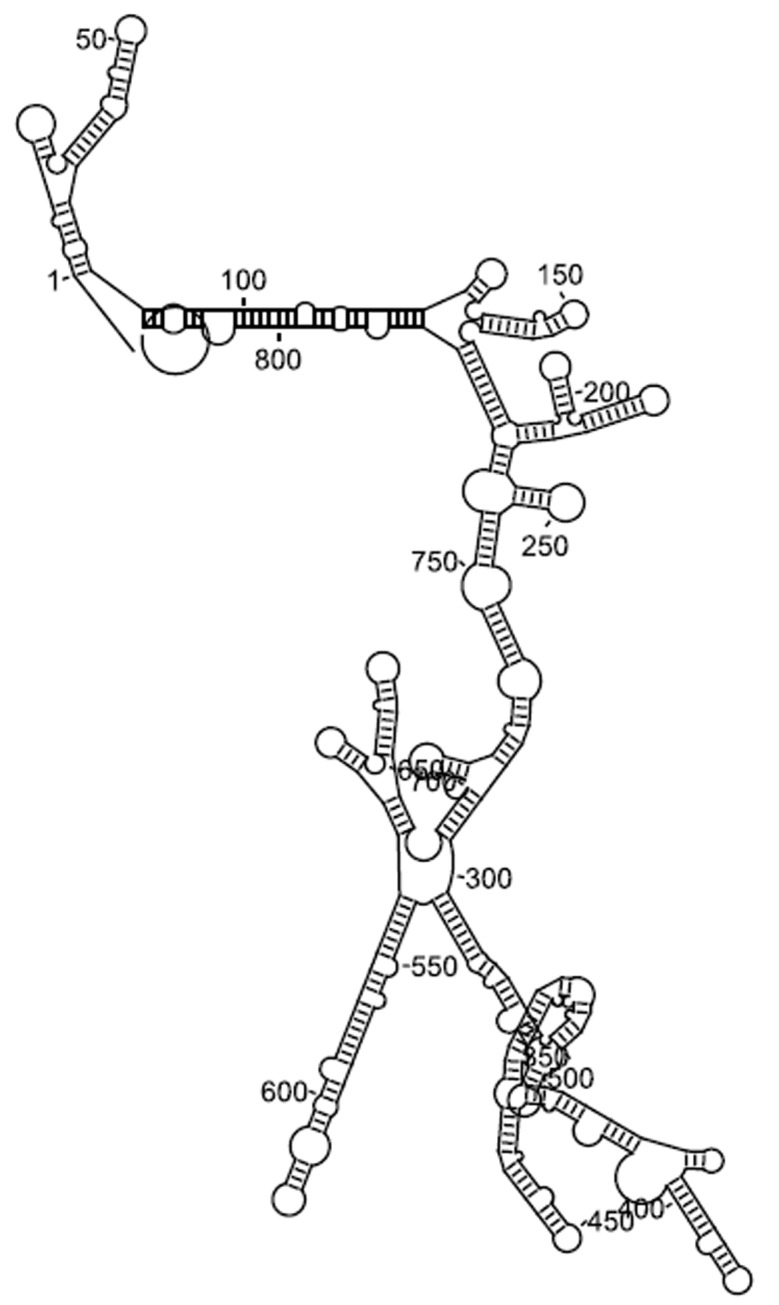
BACE1-AS secondary structure

ClustalOmega software, which has been imported in the ESPript 3.0 software for further displaying an analysis of the corresponding secondary structures (Figure 1) 29. In the ESpript output, both the secondary and primary structures are displayed in separate rows, where dots represent gaps, a stands for alpha helix, β for beta strand, TT for strict β-turns, TTT for strict α-turns, alpha helices are shown as squiggles and β-strands as arrows in the multiple alignment representation (Figure 2 - available with authors), in order to identify similarities and patterns between the proteins.

**Figure 3:**
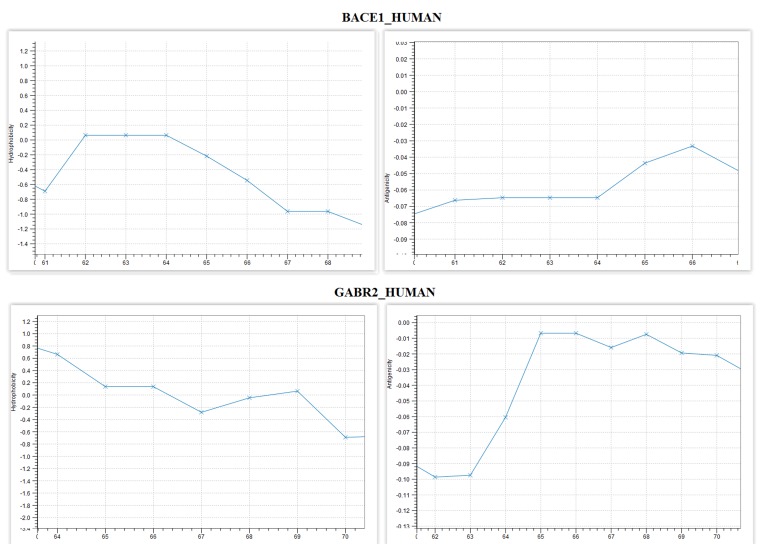
Hydrophobicity and antigenicity plots of BACE1 and GABABR2

Few interesting properties are identified in certain positions of BACE1 and GABABR2 (Table 1). In position (65) there is a decrease in hydrophobicity and a simultaneous increase in the antigenicity of BACE1 (Figure 3). In the corresponding aligned positions of (66,67), there is a decrease in hydrophobicity and antigenicity. Furthermore, in positions (64, 65) b-strict turns to occur in both proteins, while the positions (61-64) of BACE1 have the same levels of hydrophobicity and antigenicity. It is noticed from the computational analysis that β-turns are appeared to be part of the spheroproteins surface and their residues are hydrophilic 30. Therefore, it seems that in regions with β-turns hydrophobicity is reduced, affecting the folding of each protein and changing the direction of polypeptide's chain (Table 2). In this study, the regions with this interesting property can be found on the common BACE1 and GABABR2 α-turns. In positions (64,65) of BACE1 and the corresponding GABABR2 aligned positions, there are identically aligned secondary structures of β-turns. In both turns, hydrophobicity shown to be reduced from a stable state, which confirms the statements concerning the hydrophobicity. In the same region BACE1 and GABABR2 switch from positive to negative hydrophobicity (0.06 to -0.22) and (0.14 to -0.28) respectively. Furthermore, in certain β-turns BACE1 consists of aspartic acid and GABABR2 consists of lysine and glutamic acid which are hydrophilic residues. Although, the β-turns consist of different residues in general, they still affect the protein folding precisely in the same way. Several research studies since the 70s, underlie the exceptional role of β-turns while they correspond approximately to the 30% of all the protein residues 31-33. These type of secondary structures are strongly related to protein folding mechanisms depending mainly on their topology, functionality, and stability. According to their classification, β-turns can establish the initiation of folding and in some cases, the substantial destabilization of locally encoded protein features can lead to misfolding 30.

## Discussion

A secondary structure S on a sequence s is a set of ordered base pairs (Si, Sj), where i <j and si and sj represent respectively the nucleotides at positions i and j, on sequence s, that have the following properties:

i) If (si, sj) ? S then {si, sj} ?{{U,A}, {G,C}, {G,U}}, where {U,A} and {G,C} are called Watson-Crick pairs and {G,U} is called wobble pair

ii) If (si,sj) ? S and (si,sl)) ? S then j = l 

iii) If ((si, sj) ? S and (sk, sl)) ? S and i < k then l < j or j < k 

In other words, constraint i) means that only Watson-Crick and wobble ordered base pairs may form. Constraint ii) states that a nucleotide may be involved in at most one ordered base pair. Constraint iii) implies that all ordered base pairs are nested, i.e. that no pseudoknots are allowed in the secondary structure. While these constraints greatly simplify the folding algorithms, none of the above constraints is biologically relevant. Further pseudoknots appear in many important RNAs structures, albeit at a low frequency. For example, in the small ribosomal unit in E.coli from the 447 reported Watson-Crick and wobble ordered base pairs only 8 are pseudoknots 34. Any secondary structure generated under these rules can be decomposed into a unique set of a loop 35. A loop is a substructure which consists of a closing ordered base pair (Si, Sj) and all nucleotides that are accessible from this ordered base pair. A nucleotide sp is accessible from (Si, Sj) if i<p<j and there exists no other ordered base pair (Sk, Sl) in S such that i<k<p<l<j. Loops can be assigned a degree, i.e., the number of ordered base pairs in the loop and size which corresponds to the number of an unpaired nucleotide in the loop. There exist different kinds of loop depending on the amount and arrangement of their interior ordered base pairs 21. Hairpin loops have a degree of 1. Loops of degree 2 are called interior loops. Interior loops of size zero are called stacked pairs. An uninterrupted sequence of stacked pairs represents a stem. Interior loops of a size larger than 0, with adjacent interior and exterior, ordered base pairs, are called bulge loops. Multiloops are loops of degree greater than 2. Finally, exterior loops are the set of nucleotides which are inaccessible by any ordered base pair. In the literature 36 except for hairpin and interior loops, definitions for bans, multiloops, external loops, pseudoknot loops, interior pseudoknotted loops, and multi-pseudo knotted loops, can also be found. RNA secondary structures can be displayed in different kinds of representations. Depending on the use of the RNA molecules, specific representations are more or less useful. The bracket notation is a text-based representation; the structure is reflected in a string of dots and brackets. Dots denote non-bonding bases and a pair of brackets indicates a base-pair. A more convenient representation, which expands in all directions in a plane and thus is closer to spatial representation, is the squiggle plot. It is the most prominent plot to easily describe the approximate spatial structure of RNA. Ordered base pairs are given as two bases connected through either a straight line or a circle indicating the so-called wobbling base-pair G-U. Considering RNA secondary structure in a more theoretical way, the representations as trees or as arc-annotated sequences are well-accepted. Schmitt et al computed the total number of RNA secondary structures of a given length with a fixed number of ordered base pairs, under the assumption that all ordered base pairs can occur, by establishing a one-to-one correspondence between secondary structures and trees 37. In recent years, tree representations of RNA secondary structures occurred in the literature, and algorithmic applications on trees are performed successfully. For example, the full tree representation 38 associates ordered base pairs to internal nodes and unpaired bases to leaf. In a more detailed representation, each interior node is surrounded by right-most and left-most children which correspond to the 5� and 3� nucleotides of the ordered base pair, respectively. In a Shapiro-Zhang tree, the different loops and stacked regions are represented explicitly with special labels 39. Arc annotated sequences focus on representing sequences as straight lines. Arcs indicate base pairings. A similar representation to the arc-annotated sequence is the drawing of this sequence on a circle. Arcs are plotted as curved lines inside this circle. The mountain plot is useful for large RNAs. Plateaus represent unpaired regions; the heights of these mountains are determined by the number of ordered base pairs in which the partial sequences are embedded. Specifically, the mountain plot representation maps the secondary structure into a 2-dimensional graph where the x-axis represents the position along the RNA sequence and the y-axis corresponds to the number of ordered base pairs that enclose nucleotide k. The dot plot representation maps the structure to a matrix where a dot at position (i, j) represents the ordered base pair (Si, Sj). The secondary structure of an RNA molecule is the collection of ordered base pairs that occur in its 3D structure. When the 5�- end of one nucleotide fits the 3�-end of another, a p-bond is formed, while the sequence of p-bonds defines the backbone of the molecules. On the other hand certain ordered base pairs like {C, G},{ A, U}, and {G, U} form h-bonds, which cause folding of the molecular backbone into a configuration of minimal energy 40. In some cases unusual non-canonical ordered base pairs, like {G, U}, {G, A} and {C, A} replace the canonical Watson-Crick ordered base pairs, which maintained a stable helical structure. While these noncanonical pairings allow possible hydrogen-bonding interactions and can be treated as neutral evidence for a helical structure, there seems to be evidence against pairing 41. A secondary structure of size n is closed 40 if there is an h-bond connecting base 1 and n and for known integers n = 2, l = 0, there are S(l) (n-2) secondary structures of size n and rank l, establishing also a bijection between the set of all closed secondary structures Z(l)(n) and the set of all plane trees with exactly n leaves T(l)(n).

A constraint satisfaction formulation was also used for RNA prediction problem including genetic mapping 42, physical mapping 43 and structure prediction 44. The ultimate goal of structure prediction is to obtain the three-dimensional structure of biomolecules through computation. The key concept for solving the above-mentioned problem is the appropriate representation of the biological structures. Nowadays, an increasing number of researchers have released novel RNA structure analysis and prediction algorithms for comparative approaches to structure prediction, based on the fact that closed RNA structures can be viewed as mathematical objects obtained by abstracting topologically non-relevant properties of planar folding of singlestranded nucleic acids. There are a lot of approaches on this topic, such as dynamic programming algorithms 45, stochastic algorithms such as Bioambiens calculus 46, comparative methods 47, simulated annealing 48, artificial neural net algorithms and most recently evolutionary algorithms which attempt to mimic the natural folding pathway by using populations based approach 49.

## Conclusion

While specific lncRNAs have been already correlated to certain AD lesions, a new computational analysis of the proteins BACE1, Rad18, GABABR2 and hnRNPQ have been presented in this study. Using the QIAGEN CLC Main Workbench, the ClustalOmega software and the ESPript 3.0 software, a detailed analysis of the corresponding secondary structures for the sequences 6EJ3, 4F12, 4UX8, 2Y43 has been executed. The results of our computational analysis identified common properties in aligned positions with high similarity score, identical secondary structure match, increased hydrophilicity, and negative antigenicity, revealing simultaneously strong evidence that the proteins under consideration, may have common functionality in those regions that regulate folding and aggregation and prevent binding of immune factors. These conclusions reveal the significance of the most known α-turn-forming residues, which participate in ligand binding, molecular recognition, protein-protein or protein-nucleic acid interactions and modulation of protein functions and intermolecular interactions, in proteins commonly linked to AD development or progression.

## Conflict of Interest

Authors declare no conflict of interest.

## Figures and Tables

**Table 1:** Positions of interest with similar properties between BACE1 and GABABR2

Positions	61	62	63	64	65	66
BACE1_HUMAN	V	E	M	V	D	N
Positions	65	66	67	68				
GABR2_HUMAN	T	K	E	V				

**Table 2:** The numbers correspond to BACE1. In the case of a gap in BACE1, the number corresponds to GABABR2 with an additional (*). If there is a gap in both BACE1 and GABABR2, the number corresponds to hnRNPQ with an additional identifier

Regions of interest	Proteins	Structure	High similarity
64-65	BACE1_HUMAN GABR2_HUMAN	Strict β-turn	65
		Strict β-turn	
67	BACE1_HUMAN	Start of β1	67
	HNRPQ_HUMAN	Start of α1	
77-81	HNRPQ_HUMAN	α2	79
	RAD18_HUMAN	η1, the start of α1(81)	
99	BACE1_HUMAN	Start of β4	
	HNRPQ_HUMAN	Start of α3	
	RAD18_HUMAN	End of α1	
106-109	BACE1_HUMAN HNRPQ_HUMAN	β-turn (107-108)	107
	RAD18_HUMAN	end of α3 (106)	
		strict α- (107-109)	109
118-120	BACE1_HUMAN GABR2_HUMAN	β-turn (119-120)	
	RAD18_HUMAN	end of β2 (119)	
		end of β1 (118)	
136*	GABR2_HUMAN	start of α3	
	RAD18_HUMAN	start of strict α-turn	
144*	GABR2_HUMAN	end of α3	
	RAD18_HUMAN	start of η2	
149	BACE1_HUMAN GABR2_HUMAN	start of strict α-turn	
		end of β4	
155-156	BACE1_HUMAN	start of β7	
	GABR2_HUMAN	end of η2(55)	
		start of α5(56)	
	HNRPQ_HUMAN	start of α4	
	RAD18_HUMAN	β3	
173	BACE1_HUMAN GABR2_HUMAN	end of β-turn	
		end of α5	
178	BACE1_HUMAN GABR2_HUMAN	start of β8	
	HNRPQ_HUMAN	start of β6	
		start of α6	
211	BACE1_HUMAN GABR2_HUMAN	start of β9	
		end of β7	
216	BACE1_HUMAN GABR2_HUMAN	end of β9	
		start of α7	
237-240	BACE1_HUMAN GABR2_HUMAN HNRPQ_HUMAN	end of β10 (237)	237
	RAD18_HUMAN	end of β8 (239)	239
		end of α6 (240)	240
		β-turn (238-239)	
242	BACE1_HUMAN GABR2_HUMAN	start of η3	
		start of α8	
269	BACE1_HUMAN GABR2_HUMAN	end of β12	
		start of β-turn	
270	BACE1_HUMAN GABR2_HUMAN	start of β-turn	
		end of β-turn	
273-274	BACE1_HUMAN GABR2_HUMAN	end of β13 (273)	
		start of α10 (274)	
285-286	BACE1_HUMAN GABR2_HUMAN	start of β14 (286)	
		end of α10 (285)	
311-312	BACE1_HUMAN GABR2_HUMAN	end of α2 (312)	
	HNRPQ_HUMAN	end of α11 (311)	312
		start of β-turn (312)	
326-327	GABR2_HUMAN	end of β11 (326)	326
		start of β-turn (327)	327
	HNRPQ_HUMAN	end of α7 (327)	
330-331	BACE1_HUMAN	end of β16 (330)	331
	HNRPQ_HUMAN	start of η1 (331)	
420*-421*	GABR2_HUMAN	end of β-turn (420*)	
		end of η1 (420*)	
	HNRPQ_HUMAN	start of β13 (421*)	
422*-423*	BACE1_HUMAN GABR2_HUMAN	start of β-turn (423*)	422
		end of β13 (422*)	
334-335	BACE1_HUMAN GABR2_HUMAN	end of β-turn (334)	334
		start of β14 (335)	335
353	BACE1_HUMAN GABR2_HUMAN	start of β-turn	
		end of β15	
360-361	BACE1_HUMAN GABR2_HUMAN	end of β18 (361)	360
	HNRPQ_HUMAN	end of β16 (361)	361
		end of β-turn (360)
